# Patient profile, management, and quality of life associated with Dravet syndrome: a cross-sectional, multicentre study of 80 patients in Spain

**DOI:** 10.1038/s41598-023-30273-z

**Published:** 2023-02-27

**Authors:** Antonio Gil-Nagel, Rocío Sánchez-Carpintero, Vicente Villanueva

**Affiliations:** 1 Department of Neurology, Epilepsy Program, Ruber International Hospital, Madrid, Spain; 2 Pediatric Neurology Unit, Department of Pediatrics, Clínica Universidad de Navarra, Av. de Pío XII, 36, 31008 Pamplona, Spain; 3 Refractory Epilepsy Unit, Department of Neurology, University and Polytechnic La Fe Hospital, Valencia, Spain

**Keywords:** Paediatric research, Epilepsy, Quality of life

## Abstract

The aim of this study was to describe the profile of patients diagnosed with Dravet syndrome (DS), their clinical management, and the impact of DS on their quality of life (QoL) and family. Data of 80 patients from 11 centres in Spain was collected. Patients (47.5% female) were 12.7 (9.6) years on average (SD, standard deviation). Despite the first episode occurred when patients were a mean (SD) of 0.4 (0.2) years, DS was not diagnosed until they were 6.9 (10.1) years old. The majority (86.7%) had *SCN1A* gene mutations and 73.4% had seizures during the last year (mostly generalized motor seizures [47.8%]). The mean (SD) number of status epilepticus episodes was 3.6 (8.0) since diagnosis and 0.1 (0.5) in the last year. On the Health Utilities Index Mark (HUI) multi-attribute scale, the mean global score (SD) was 0.56 (0.24) in HUI2 and 0.32 (0.37) in HUI3. The impact of the disease was severe in most patients (HUI2, 81%; HUI3, 83.5%). In the Care-related QoL (CarerQol) the mean (SD) well-being score was 7.2 (2.1). Most caregivers (90%) were satisfied with their caregiving tasks, although 75% had difficulties combining these tasks with daily activities, 68.8% reported mental health problems and 61.2% physical problems.

## Introduction

Dravet syndrome (DS) is a developmental and epileptic encephalopathy characterized by recurrent seizures and neurodevelopmental impairment. Around half and one-third of patients with DS present with a tonic–clonic or focal clonic (hemiclonic) seizure, respectively, which are frequently febrile or vaccine-related^1,2^. Subsequent seizures are often fever-related, although spontaneous seizures or seizures provoked by other stimuli can also occur. Seizures can be generalized, of focal onset or of unknown onset and after the first year of life, different seizure types can appear such as myoclonic, atypical absences, focal impaired-awareness seizures or more rarely atonic.

The disease evolves with age and motor, behavioural, and cognitive impairments of variable severity appear after seizure onset^3^. The incidence of DS has been reported to be 1 in 15,000 to 1 in 40,000^4,5^. The majority of patients are diagnosed in their childhood^6^, and initial symptoms occur during the first year of life^1,7^. Children with DS face a substantial risk of early death^5,8^, commonly caused by sudden unexplained death in epilepsy and status epilepticus^5,8,9^. Frequent seizures, associated comorbidities, and the uncertainty of fatal outcomes have a negative impact not only on the health-related quality of life (HRQoL) of the patients but also on their caregivers^10–12^. As expected, seizure-related-events, hospitalizations, and in-home medical care impose an important economic burden to the healthcare system^12^.

The majority of DS cases have variants in the *SCN1A* gene*,* which codes for a neuronal voltage-gated sodium-channel alpha-subunit^13^, and the nature of the variants can affect the phenotype^14^. Additional genes have also been identified with DS phenotypes^15^. These phenotypes are considered as DS with an alternative genetic cause by some experts and as encephalopathies mimicking DS or DS-like by others^15^. Genetic testing has considerably helped to confirm the diagnosis of the disease, which was previously based only on clinical assessment^6^. A clear understanding of the patient profile and an early diagnosis are crucial for many reasons such including, among other , to avoid using sodium channel blockers that are contraindicated for DS as they may exacerbate seizures and worsen prognosis^5,16,17^; and to incorporate indicated treatments that have showed to be effective to control seizures and comorbidities, such a stiripentol, cannabidiol, and fenfluramine^17^.

Data on the characteristics of DS patients, and the impact of the disease on the patients and their families is still limited^10,18–21^. In the case of Spain, published data were collected before the newer treatments for DS were approved, and patients HRQoL was not assessed^17^. The present study aimed to describe the demographic and disease-specific clinical profile of patients diagnosed with DS in Spain, and to expand knowledge of their clinical management, HRQoL of the patient and the family, and healthcare resource use, by obtaining data from clinical records and patient-reported outcomes (PROs) at the study visit.

## Methods

### Study design, setting, and participants

This was a national, multicentre, cross-sectional, observational study conducted in the Departments of Neurology or Paediatrics of 11 centres in Spain. All the participating sites were referral centres for DS in Spain. Data were collected from patient’s medical records and from questionnaires completed at the study visit. Study visits occurred from October 2020 to March 2021 and coincided with any of the routine follow-up visits to the specialist.

The selection criteria were patients (1) diagnosed with DS according to the ILAE criteria^22^, (2) who attend to Departments of Neurology or Paediatrics of the participating centres for DS management, and (3) have signed the informed consent (patients or their legal representatives). When a patient with DS was identified, the patient or the patient’s caregivers were invited to participate in the study and were required to sign the informed consent form before their inclusion in the study.

The study complied with the ethical principles contained in the Declaration of Helsinki, with Good Pharmacoepidemiology Practices, and with local regulations. It was approved by the Ethics Committees of four of the participating sites: Hospital Clínico San Carlos (Madrid, Spain), Hospital Universitario Virgen de las Nieves (Granada, Spain), Hospital Sant Joan de Déu (Barcelona, Spain), and Hospital Universitario y Politécnico La Fe (Valencia, Spain). The STROBE (Strengthening the Reporting of Observational Studies in Epidemiology) guidelines have been followed.

### Assessments

Demographic and clinical data were retrieved from medical records. Demographic data included age and sex. Clinical data included age at first seizure and at diagnosis; seizure number, severity, and type in accordance with 2017 International League Against Epilepsy (ILAE)^23^ (at onset, at study visit, and changes over time); status epilepticus (defined as a seizure with a duration ≥ 30 min or a series of seizures in which the patient does not regain normal mental status between seizures); results from performed tests (genetic tests, electroencephalogram [EEG], and magnetic resonance imaging [MRI]); comorbidities; previous and current treatment (antiepileptic drugs and other therapies); and healthcare resource use (number of visits to physician and admissions to the emergency room or intensive care units [ICU] and attendance to day-care or a rehabilitation centre).

Patient HRQoL was evaluated using the Health Utilities Index Mark 2 and Mark 3 (HUI2/3)^24^ and the SINDRA questionnaire at the study visit. Both questionnaires were addressed to patient’s caregivers. The HUI 2/3 consists of 15 questions evaluating the following attributes: vision, hearing, speech, ambulation, dexterity, emotion, cognition, self-care, and pain. Each attribute is assigned a score on a 0 to 1 scale, with 0 corresponding to the worst health status and 1 corresponding to the best health status. In addition, an overall HUI score from − 0.371 to 1, where 0 corresponds to death, 1 represents perfect health, and negative scores are health states considered worse than dead. The SINDRA questionnaire was created ad hoc and consisted of 17 statements: seven related to patient functional skills, eight related to patient daily activities, and two related to caregivers’ work absence or work leave caused by patient caring. The SINDRA questionnaire is provided on Additional information. The impact of caregiving on patient’s caregivers was assessed with the care-related quality of life (CarerQoL) questionnaire^25^ at the study visit. The questionnaire consists of 7 items graded as “no”, “a little” or “a lot” regarding the description of the caregiving situation (CarerQol-7D) and the valuation of informal care in terms of well-being using a visual analogue scale (CarerQol-VAS) in which 0 is “completely unhappy” and 10 “completely happy”. The Spanish validation of the HUI 2/3 and the CarerQoL-7D questionnaires were used with permission. All the information was collected in a case report form by a healthcare professional.

### Statistical methods

A sample size of 78 patients was calculated to identify the profile of patients integrated by characteristics present in 50% of the studied population, with an accuracy of ± 11 percentage units and an alpha risk of 0.05 for a bilateral analysis. A 5% data loss was assumed due to incomplete data or other causes.

A descriptive analysis was performed. Quantitative variables were described using central and dispersion-tendency statistic measures (mean, median, standard deviation [SD], and interquartile range [IQR]). Qualitative variables were described using absolute (N) and relative frequencies (valid percentages, unless specified otherwise). Missing data was not imputed and was left as lost. Analyses were performed using IBM SPSS statistics software version 22.0.

## Results

### Demographics and clinical characteristics

A total of 80 patients were examined for eligibility. All the patients met the selection criteria and were included in the study. The characteristics of the patients are shown in Table 1. Briefly, the median (IQR) age was 10.8 (6.5–14.9) years old, and 38 (47.5%) were female. The first seizure occurred at a median (IQR) age of 0.40 (0.32–0.57) years old, but patients were not diagnosed until 2.2 (0.8–5.2) years later, when they were 2.8 (1.4–7.8) years old on average. The most common comorbidities were cognitive deficits (58.2%) and motor impairments (55.2%). Mutations in the *SCN1A* gene were present in 67 (89.3%) out of 75 patients with available genetic data. Genetic alterations in other genes were also observed, but with a lower frequency. An abnormal MRI was observed in 14.3% of patients.

**Table 1:** Patient characteristics.

	Patients	N
Gender (female), n (%)	38 (47.5)	80
Age	10.8 (6.5–14.9)	
Median (IQR)	10.8 (6.5–14.9)	80
Mean (SD)	12.7 (9.6)	
Fully vaccinated (yes), n (%)	72 (91.1)	79
Age at first seizure		
Median (IQR)	0.40 (0.32–0.57)	73
Mean (SD)	0.47 (0.26)	
Age at diagnosis		
Median (IQR)	2.8 (1.4–7.8)	68
Mean (SD)	6.9 (10.1)	
Comorbidities and impairments, n (%)		
Cognitive delays	39 (58.2)	67
Motor impairments	37 (55.2)	
Orthopaedic disorder or scoliosis	18 (26.9)	
ADHD	16 (23.9)	
Sleep disorders	15 (22.4)	
Behavioural disorders	14 (20.9)	
Neurodevelopmental disorders	12 (17.9)	
Autism	11 (16.4)	
Dental alterations	7 (10.4)	
Other	16 (23.9)	
Genetic alterations, n (%)		
*SCN1A*	67 (89.3)	75
*PCDH19*	5 (6.7)	
*GABRG2*	2 (2.7)	
*SCN8A*	2 (2.7)	
*SCN9A*	2 (2.7)	
*SCN2A*	1 (1.3)	
*CH2D*	1 (1.3)	
*STXBP1*	1 (1.3)	
*KCNA2*	1 (1.3)	
*SCN1B*	1 (1.3)	
*GABRA1*	1 (1.3)	
*KCNQ3*	1 (1.3)	
*SLC6A11*	1 (1.3)	
No genetic alteration found	4 (5.3)	
EEG, n (%)		
Normal*	29 (36.7)	79
EEG slowing	27 (34.2)	
Focal epileptiform activity	23 (29.1)	
Generalized epileptiform activity	21 (26.6)	
MRI, n (%)		
Normal	66 (85.7)	77
Abnormal	11 (14.3)	

*A Interictal EEG without pathological abnormalities (neither epileptiform abnormalities nor slow activity).

**Figure 1:**
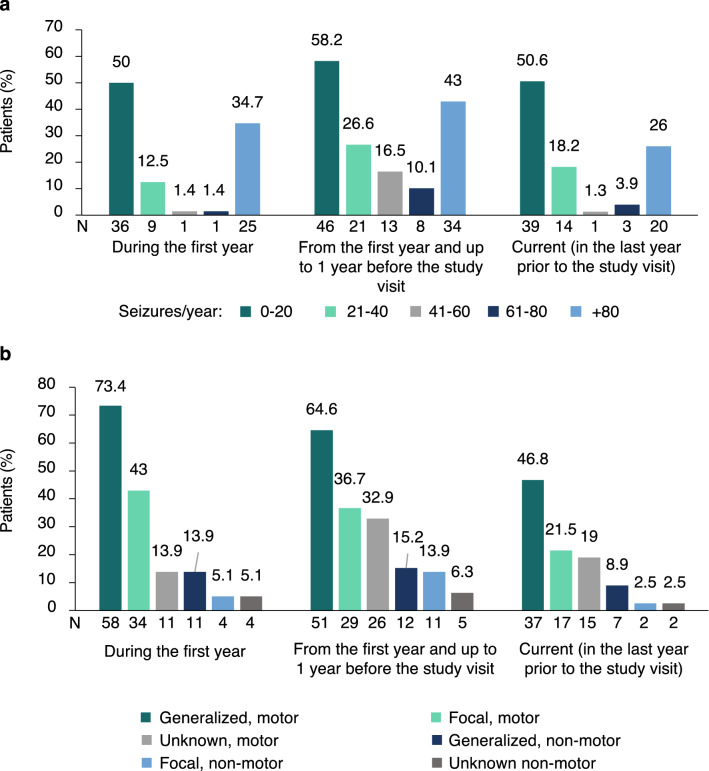
Number and type of seizures during the disease course (all patients). In both figures, percentages are showed for three time periods: during the first year after diagnosis, from the first year after diagnosis to the year before the study visit, and during the year before the study visit. Note that the percentage of patients from the first year after diagnosis to the year before the study visit (in both figures) sum more than 100% because records for several years per patient have been included here. (**a**) Seizure frequency grouped by categories (from 0 to 20 seizures/year, from 21 to 40 seizures/year, from 41 to 60 seizures/year, from 61 to 80 seizures/year, and more than 80 seizures or uncountable/year) for the three time periods. (**b**) Seizure type during the first year after diagnosis, from the first year after diagnosis to the year before the study visit, and during the year before the study visit for the three time periods. Focal to bilateral tonic–clonic seizures with rapid propagation and primary bilateral tonic–clonic seizures were considered under the same type of bilateral tonic clonic seizures.

The number of patients with seizures during the first year of diagnosis was 79 (100%), 73 (92.4%) from the year after diagnosis until one year before the study visit, and 58 (73.4%) on the year before the study visit. Half of the patients had ≤ 20 seizures during the first year (n = 36; 50%) and during the year before the study visit (n = 39; 50.6%). The number of patients with ≥ 80 seizures was 25 (34.7%) the year after the diagnosis and 20 (26%) the year before the study visit (Fig. 1a). Generalised motor seizures were the most frequent type of seizures and represented the 47.8% of all registered seizures, followed by motor focal (21.6%), generalized nonmotor (15.2%), motor of unknown onset (8.0%), nonmotor of unknow onset (4.6%), and focal nonmotor (2.8%). Different types of seizures and triggers were observed within the same patient. Figure 1b shows the different types of seizures that occurred over different periods of the disease course. Seizures were usually triggered by temperature (81.6%), lights (14.5%), sounds (3.9%), and other triggers (73.7%). The number of seizures remained stable in 33 (47.1%) patients, decreased in 21 (30%) patients, and increased in 16 (22.9%) patients from the first year after diagnosis to the year before the study visit; the change in the number of seizures per year was not statistically significant (p = 0.150).

The history of status epilepticus is showed in Table 2. Since diagnosis, the median (IQR) number of status epilepticus was 2.0 (1.0–3.8) and 0.0 (0.0–0.0) episodes for the last year before the study visit (these include status epilepticus that might have occurred at disease onset before adequate medication was initiated).

**Table 2:** History of status epilepticus.

	Patients
Patients with available data on episodes since diagnosis (n)	68
Median (IQR)	2.0 (1.0–3.8)
N (%)	
0	14 (20.6)
1–5	45 (66.2)
6–10	5 (7.4)
11–20	2 (2.9)
> 20	2 (2.9)
Patients with available data on episodes during last year (n)	76
Median (IQR)	0.0 (0.0–0.0)
N (%)	
0	67 (88.2)
1	5 (6.6)
2	3 (3.9)
3	1 (1.3)

### Previous and current treatment

Seventy-five patients (93.8%) had received at least one prior treatment for DS, with a mean number of 4.6 (3.9) previous treatments per patient. The most common prior treatment was levetiracetam (62.7%) followed by topiramate (37.3%). Seventy-nine patients (98.8%) were receiving treatment for DS at the study visit, with a mean number of 3.2 (1.3) treatments per patient; the most prevalent were sodium valproate (86.1%) and clobazam (62.0%) (see Table 3). Additionally, fifteen (18.8%) patients were receiving their current treatment as part of a clinical trial (12 were treated with fenfluramine, 2 with cannabidiol, and 1 with soticlestat). Rescue medication was needed in 36 (45.0%) patients (21 received midazolam, 18 diazepam, 2 levetiracetam, 1 lorazepam, and 1 clonazepam). The route of administration of rescue medication in these 36 patients was rectal (17 patients), oral (13), buccal (6), intravenous (5), intranasal (3), subcutaneous (1), and intramuscular (1).

**Table 3:** Current and prior treatment (taken in > 2% of all patients).

	Patients	
	Current	Prior
Pharmacologic	N (%)	
Sodium valproate	68 (86.1)	18 (24.0)
Clobazam	51 (64.6)	17 (22.7)
Topiramate	26 (32.9)	28 (37.3)
Stiripentol	21 (26.6)	19 (25.3)
Levetiracetam	14 (17.7)	47 (62.7)
Cannabidiol	8 (10.1)	8 (10.7)
Brivaracetam	7 (8.9)	2 (2.6)
Lamotrigine	5 (6.3)	17 (22.7)
Zonisamide	5 (6.3)	19 (25.3)
Fenfluramine	5 (6.3)	3 (4.0)
Clonazepam	4 (5.1)	18 (24.0)
Ethosuximide	4 (5.1)	5 (6.6)
Lacosamide	4 (5.1)	11 (14.7)
Diazepam	3 (3.8)	–
Carbamazepine	2 (2.5)	14 (18.7)
Phenobarbital	2 (2.5)	19 (25.3)
Oxcarbazepine	–	11 (14.7)
Vigabatrin	1 (1.2)	7 (8.2)
Perampanel	1 (1.2)	7 (8.2)
Phenytoin	–	6 (7.9)
Primidone	–	4 (5.3)
ACTH	–	3 (3.9)
Pyridoxine	–	3 (3.9)
Non pharmacologic		
Ketogenic diet	4 (5.1)	8 (10.7)
Vagus nerve stimulation	3 (3.8)	1 (1.3)
l-carnitine	3 (3.8)	–

### Health-related quality of life

**Figure 2:**
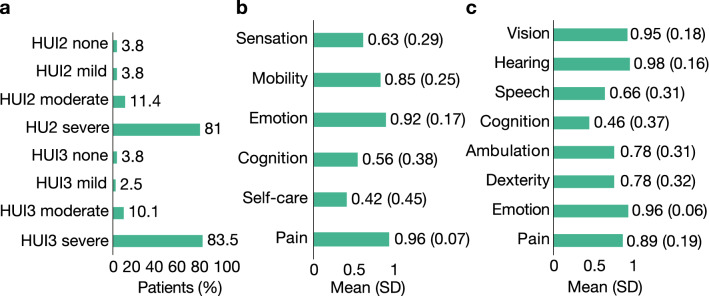
HUI2/3 scores (**a**) Mean (SD) multiatribute score in the HUI2/3 per categories (HRQL alteration: none, mild, moderate, severe); the figure shows the percentage of patients within each category (**b**) Median scores of the single-attribute utilities in the HUI2 (**c**) Mean (SD) scores of the single-attribute utilities in the HUI3.

The HUI2/3 was replied in 74 patients by their mother or father, in four patients by a healthcare professional, in one patient by her/his partner, and in another patient by an unspecified responder. The mean (SD) overall multi-attribute score was 0.56 (0.24) in the HUI2 and 0.32 (0.37) in the HUI3. The impact of the disease was severe on most patients in the HUI2 (81%) and HUI3 (83.5%). Figure 2 represents HUI2/3 scores.

The CarerQol was replied by all patients’ caregivers (n = 80) (Table 4). The mean (SD) CarerQol-VAS score was 7.2 (2.1). Most caregivers (90%) felt some/a lot of fulfilment and 58.7% received some/a lot of support. A lot of problems combining daily activities with care-tasks and mental health problems were reported by 33.8% and 22.5% of patients’ caregivers, respectively.

**Table 4:** CarerQoL scores.

	Patients (n = 80)		
CarerQol-VAS score, mean (SD)	7.2 (2.1)		
CarerQoL-7D, N (%)	No	Some	A lot
Fulfilment	8 (10.0)	18 (22.5)	54 (67.5)
Relational problems	52 (65.0)	21 (26.3)	7 (8.8)
Mental health problems	25 (31.3)	37 (46.3)	18 (22.5)
Problems with daily activities	20 (25.0)	33 (41.3)	27 (33.8)
Financial problems	41 (51.3)	25 (31.3)	14 (17.5)
Support	33 (41.3)	27 (33.8)	20 (25.0)
Physical health problems	31 (38.8)	33 (41.3)	16 (20.0)
CarerQoL-VAS, mean (SD)	7.2 (2.1)		

The SINDRA questionnaire was partially completed by all patients’ caregivers and fully completed by 76. Scores for the SINDRA questionnaire regarding to the patients’ and caregivers’ situation are showed on Table 5.

**Table 5:** SINDRA questionnaire scores.

	Patients
Functional skills, N (%)	80 (100)
The patient needs a wheelchair	19 (23.8)
The patient is dependent for eating	48 (60.0)
The patient is unable to communicate or communicates inconsistently	32 (40.0)
The patient does not communicate with words	18 (22.5)
The patient manipulates objects with the hands	70 (87.5)
The patient has vision problems	11 (13.8)
The patient goes to school regularly	66 (82.5)
Daily activities, N (%)	76 (100)
The patient get dressed herself/himself	25 (32.9)
The patient uses knife and fork	31 (40.8)
The patient brushes her/his teeth	42 (55.3)
The patient washes her/his hands	50 (65.8)
The patient drinks by herself/himself	64 (84.2)
The patient is able to scribble with a pen or pencil	67 (88.2)
The patient uses an electronic device	60 (78.9)
The patient can walk alone without help	58 (76.3)
Situation of the caregiver, N (%)	79 (100)
Had to take time off from work to take care of the patient	52 (65.8)
Number of days off last year	50 (100)
Mean (SD)	59.5 (102.7)
Median (IQR)	16.0 (4.5–55)
Had to quit my job to care for the patient	38 (48.1)

### Healthcare resource use

Among those with available data (n = 62), most patients (n = 51) visited the physician in the year before the study visit, with a mean of 7.9 (8.4) visits. During the same period, 20 patients were admitted to the emergency room and three patients to the ICU. Details on healthcare resource use are showed on Table 6.

**Table 6:** Healthcare resource use.

	Patients
Visits to physician in the last year (yes), n/N* (%)	51/62 (82.3)
Number of visits, mean (SD)	7.9 (8.4)
Number of visits, median (IQR)	5.0 (3–9)
Admissions in the last year (yes), n/N* (%)	23/80 (28.8)
Emergency room, n (%)	20 (87.0)
ICU, n (%)	3 (13.0)
Duration of admissions to the emergency room (days), n	18
Mean (SD)	3.4 (3.3)
Median (IQR)	2.5 (0.7–5.6)
Duration of admissions to the ICU (days), n	2
Mean (SD)	11.5 (10.6)
Median (IQR)	11.5 (4.0)
Attendance to day-care centre/rehabilitation centre (yes) n/N (%)	36/79 (45.6)

*N* number of patients with data available for that variable, *n* number of patients with data available for that variable, excluding those where ‘0’ was reported.

## Discussion

This cross-sectional study provides updated data on the profile, management, and HRQoL of patients with DS, as well as the impact of the disease on their families. The study sample included 80 patients, which is around 15–23% of the total population with DS in Spain^6^. Thus, the sample of the present study represents a broad coverage of these patients and their families in Spain.

The study confirmed a substantial delay in a definite diagnosis. On average, patients were diagnosed almost 7 years after their first seizure, which is a longer period than the one previously reported in Spain^6,21^, other European countries^20^, and the US^26^. This diagnosis delay could be partially explained by the average age of our sample, who was slightly higher compared to prior studies^6,20,21,27^. Indeed, another study conducted in Spain showed that older patients had longer diagnostic delays than younger patients^21^. Older patients might have experienced more difficulty in accessing timely genetic tests, as tests were not clinically available in Spain until 2003^6^. Moreover, knowledge about DS has significantly increased after the discovery of the *SCN1A* gene as the cause of more than 80% of DS cases. Therefore, diagnostic delays could have been caused by poor education of clinicians on the syndromic phenotypical spectrum of DS and low availability of specialized epilepsy centres in the previous decades. Prompt diagnosis is key to avoid using contraindicated drugs and to start new indicated treatments as soon as possible to, in turn, improve the prognosis^28,29^. A considerable proportion of our patients had been previously treated with contraindicated drugs (i.e., sodium channel blockers: carbamazepine, lacosamide, lamotrigine oxcarbazepine, and phenytoin), which might have exacerbated seizures, increased the risk of status epilepticus, and worsened cognitive outcomes^30,31^.

DS was associated with wide-ranging comorbidities, including cognitive deficits, motor impairments, orthopaedic disorders or scoliosis, ADHD, and sleep and behavioural disorders. In line with previous observations^21,32^, cognitive deficit was the most prevalent comorbidity. Interestingly, the prevalence of cognitive and motor impairments as well as sleep disorders here was slighter lower compared to prior observations from surveys^11,18,20,21,27,33^. This apparent discrepancy could be explained by the fact that the prevalence of health conditions seems to be lower when recorded by clinicians on medical records (like in our study) than when self-reported by young adults in surveys (like in the above-mentioned studies)^34^. Higher prevalence of comorbidities in survey-based studies could be due to overestimation of the comorbidities by respondents in surveys, or by an underdiagnosis of the conditions in the clinical practice. Additionally, it could also be explained by the inclusion of patients with less severe DS.

Regarding genetics, most patients had genetic tests conducted, according to current recommendations^35^. As expected, the most prevalent genetic alteration was in the *SCN1A* gene. Other genes associated with DS previously described^15,36^ were also detected in our patients. The discussion on whether different genes cause DS or whether they give rise to distinct encephalopathies^37,38^ is still open. As diagnose of DS is improving, more DS-like phenotypes are being identified, such as encephalopathies related to *PCDH19* pathogenic variants^39^. Studies linking the genetic and phenotypic profile are warranted to throw light on this question, informing diagnosis and therapeutic approaches, and allowing the implementation of precision medicine.

Almost 80% of patients had at least one episode of status epilepticus over their disease. A previous study that included 241 cases with DS with *SCN1A* mutations, also reported the same percentage of patients presenting episodes of status epilepticus^5^. Status epilepticus has been shown to predict worse developmental outcomes, lead to emergency room admissions, and to be life-threatening^5,27^, but they are potentially preventable. The proportion of patients without any status epilepticus in the year before the study visit was high (88.2%). The reduction of status epilepticus in the year before the study visit could be explained by an improvement in patients’ management (earlier diagnosis in the last year and better use of rescue medication). Also, as the rate of status epilepticus usually declines with age, older DS patients might contribute to the low rate of status epilepticus observed in this cohort in the year before the study visit.

Our study also showed that the presence of any type of seizures was considerable among patients, with nearly three-quarters of them having at least one seizure the year before the study visit. This number duplicates the frequency previously reported in Spanish patients^27^. In terms of antiseizure medication, sodium valproate, followed by clobazam, were the most frequently current treatment used, in line with observations from prior studies^5,40^. Moreover, almost half of our patients (45%) needed rescue medication. Even if this proportion is lower than in previous studies (90%)^40^, this number is still considerable^40^.

Despite the fact that most patients were treated with the recommended antiseizure drugs^35^, an important proportion of them remained drug resistant, reinforcing the need of new therapies for DS. The recently approved adjunctive therapies, cannabidiol and fenfluramine, have showed efficacy in reducing the frequency of seizures in DS and are well-tolerated^17,28^. In this study only a small number of patients were already receiving these therapies. In the future, more patients are expected to receive these treatments, hopefully resulting in better seizure control^41^.

Results from the HUI2/3 showed that the impact of the disease on patient HRQoL was severe in more than 80% of the patients. The HUI has been used to measure the HRQoL in patients with a wide range of chronic disease, including epilepsy^42,43^, but, as far as we know, not in patients with DS. Patients had an average overall multi-attribute score of 0.32 in the HUI3 (closest to 0 [death] that to 1 [perfect health]). This score is similar to that reported in patients with tuberous sclerosis complex and epilepsy (0.33)^43^ and considerably lower than in the general population within a similar age range (0.80–0.92)^44^. The attributes that were more altered were sensation and self-care (HUI2), and cognition and speech (HUI3). Most respondents reported relatively high scores for the vision, hearing, emotion, and pain dimensions. High scores in the emotion dimension suggests that most patients adjusted to their condition, and that the families might have strategies and tools to limit the physical and psychological impact of the disease. Results from the SINDRA questionnaire confirmed alterations on functional skills, including communication and mobility, and daily activities in an important number of patients, in line with results of studies from other countries^11,45^.

Scores on the CarerQoL indicated that, even though most carers felt some or a lot of fulfilment by caring for the patient, having a child with DS negatively affected their daily activities, mental health, physical health, relations, and/or economy. Other studies^11,45–48^ also reported a negative impact on the HRQoL, emotional wellbeing, daily activities, relationships, and finances of the carers, but they used generic measures of HRQoL^47–50^ or qualitative interviews^11,45^. Instead, we used a measure specifically developed to assess HRQoL that includes the negative and positive effects of caregiving, providing a holistic assessment of the impact of caregiving and broadening the evidence. Our study also showed that the impact of caring on the ability to work is high. Almost half of the carers had to quit their job and 65% of them took time off from work to care for the patient. A substantial impact on work productivity had been previously reported^47^, with lost productivity resulting in high indirect costs and financial burden^48,49^. Indeed, financial problems were highlighted as an issue by half of the carers in our study, and 25% of them indicated a lack of social and family support when needed. Although we did not analyse the results separately by carers’ gender, other studies have shown that mothers had more lost work days and reduced working hours than fathers^49^ and had a greater impact on their professional and social life^33^. Overall, these findings suggest that supportive services for DS families are an unmet need for DS management in Spain.

The availability of cross-sectional data on seizures, symptoms, HRQoL, and healthcare resource use, such as the findings provided here, are of great importance for understanding the current situation of DS patients and their families and for improving their management. A recently published study conducted in 75 patients with DS in Germany has shown the utility of composite symptom scores based on physical (symptoms), psychosocial (HRQoL) and care requirement (disability and resource use) domains^50^. These scores were strongly associated with seizure measures (seizure-free intervals, seizure-free days and total seizures), suggesting that the prolonged remission of seizures is associated with improved physical and psychological functioning. Here, we did not analyse the potential associations between seizures and non-seizure manifestations, and future studies addressing this hypothesis are warranted. Also, studies assessing the effectiveness of treatments for DS should not only consider the reduction in seizure frequency but improvements in the rest of the affected domains as well.

The present study had some limitations. First, although diagnosis of DS remains a clinical diagnosis, no strict criteria were established among researchers for patient inclusion in the study. This could have led to some variation in phenotypes included in the study. Second, the sample studied was limited to patients followed in referral centres from Spain, and this might not reflect the overall situation of DS patients and might have introduced a sample selection bias; patients followed in general neurology clinics might have different disease severity or HRQoL. Nevertheless, the high proportion of patients included in the study regarding the total population of DS patients in Spain allows to generalise the results to the DS population at large. Third, no control group (for instance patients with other type of epileptic encephalopathy) was included in the study, and thus it is possible that the results obtained were not specific to DS. Forth, the study did not address phenotype-genotype associations, or the impact of the epilepsy variables recorded on the measures of QoL, which are of great relevance and should be analysed in future studies. Finally, due to the absence of a validated and specific questionnaire to evaluate HRQoL in DS, a generic and an ad-hoc questionnaire were used here. Cognitive debriefing and psychometric assessment of the SINDRA questionnaire were not conducted and therefore, the results of this PRO must be taken with caution. This is a limitation that all studies assessing HRQoL in DS have, and that should be addressed once such tools are developed.

## Conclusions

Our study provided data on the profile and management of 80 patients with DS and on the HRQoL of these patients and their families. Patients in Spain are, on average, 12.7 years old, have a significant delay in the diagnosis, and suffer a wide range of comorbidities (mainly cognitive deficits and motor impairments). The most common current treatment was sodium valproate, and some patients were receiving treatments indicated for DS (stiripentol, cannabidiol, and fenfluramine). The impact of the disease was severe on most patients, with a considerably alteration in functional skills and daily activities. Despite most caregivers were fulfilled by their role, the disease had a negative effect on their daily activities, health, relations, and work. Further research is required to assess the influence of certain factors such as advances in genetic diagnoses and use of newer treatments on improving disease severity and the HRQoL of these patients and their caregivers.

## Data Availability

The datasets generated during and/or analysed during the current study are available from the corresponding author on reasonable request.
